# Evidence-based assessment of new technologies: good for Barack Obama's healthcare plan and good for Brazil, and at everyone's reach through HTAi Rio 2011

**DOI:** 10.1590/S1516-31802010000300001

**Published:** 2010-05-06

**Affiliations:** I IMD, MSc, PhD. Director of the Brazilian Cochrane Center. Editor-in-chief of the São Paulo Medical Journal and the journal Diagnóstico & Tratamento of the Associação Paulista de Medicina. Titular professor of Evidence-Based Medicine and Emergency Medicine of Universidade Federal de São Paulo — Escola Paulista de Medicina (Unifesp-EPM).

To evaluate a type of technology either within medicine or within the broader field of healthcare, the best scientific evidence needs to be mapped out with regard to the efficacy (studies under ideal conditions), effectiveness (in the real world), efficiency (taking into account practicality and costs) and safety of each approach. These requirements are important for physicians’ decisions in relation to patients, for the clientele of private healthcare, for health ministries’ decisions on healthcare policies in major countries and for the World Health Organization. Such assessments require impartiality, seriousness, competence and respect for the quality criteria of scientific evidence, taken into account during decision-making processes.

Recently, the members of the scientific committee of Health Technology Assessment International (HTAi) met in Rio de Janeiro to define the scientific content of the international congress that is to be held in Rio de Janeiro between June 25 and 29, 2011. The theme of the congress will be the use of “health technology assessments based on scientific evidence to enable sustainability of healthcare systems”. It is well known today that erroneous decisions to incorporate products or interventions without proving their efficiency and safety may lead to great harm to patients, bankruptcy of healthcare systems and tarnishing of the reputations of the healthcare professionals who risked their credibility by choosing approaches for which there was no evidence of greater efficacy. At the scientific committee's meeting in Rio de Janeiro, in April 2010, supported by the Brazilian Ministry of Health, the preliminary guidelines for the 2011 event were defined, in accordance with the following text.

## Health technology assessment for Healthcare System Sustainability

Following discussions at the United Nations Conference on Environment and Development in Rio in 1992 (ECO-92) and in subsequent global meetings, sustainability is now seen as the key issue facing mankind in the 21^st^ century. Much of the debate has been around the sustainability of the climate and environment, but the sustainability of human systems also presents significant challenges. The HTAi 2011 Annual Meeting in Rio will focus on the sustainability of healthcare systems and the role of health technology assessment (HTA) in supporting this.

Demands on healthcare systems are increasing as populations grow and/or age, science advances and public expectations of healthcare and quality of life increase. Some new technologies address previously untreatable conditions, but often at high cost, and many of the main causes of global ill-health still lack effective treatments. Quality, equity and universal coverage and access are of increasing concern in low and middle income as well as high income countries. At the same time, spending on healthcare is coming under increasing pressure and scrutiny.

A key component of sustainability is the appropriate use of technology and the allocation of resources to maximize the value obtained. HTA aims to provide all who make decisions with the information they need to do this in an evidence-based, scientifically valid, transparent and accountable way. HTA is thus critical for the sustainability of healthcare systems.

HTA is increasingly being called upon by healthcare systems to support improvements in quality and coverage and to help them move towards sustainability. A number of HTA systems have developed around the world and have made considerable progress, and other such developments are planned. But HTA as it is currently practiced and used is not yet responding adequately to some of the key needs of healthcare system decision makers, particularly in primary care, health promotion, healthcare system issues and public health. The science and practice of HTA need to evolve to tackle these challenges.

HTAi 2011 will address these and other key issues facing HTA through a range of parallel panel, workshops, oral and poster sessions, and three high profile Plenary Sessions:

### 1. The challenge: Healthcare system sustainability in the 21^st^ century

What are the challenges facing healthcare systems in the 21^st^ century? How do these vary between high, middle and low-income countries? What approaches are being taken to ensure the sustainability of healthcare systems? What is the role of HTA in sustainability?

### 2. Progress: Case studies of HTA development and impact on healthcare systems

How is HTA developing and being used in countries within and beyond the Americas? What models have been developed and what impact are they having on healthcare delivery and health, and on wider issues such as costs, equity and access? Does it vary according to the healthcare system (e.g. private versus public)?

### 3. Next steps: Developing HTA for sustainable healthcare systems

How does HTA need to evolve to provide better support for the sustainability of healthcare systems? What role can and should it play in primary care, public health and healthcare system issues? What role can and should it play in low and middle-income as well as in high-income countries? How can the culture of evidence-based decision making be promoted across society? What actions need to be taken to support these developments in HTA?”

The congress will be an excellent opportunity to exchange knowledge about how to make decisions based on quality evidence, going from the individual level (physicians and patients) to the collective level (healthcare policies).

In an interesting article on this topic, it was reported that President Barack Obama's new healthcare plan envisages investments of one billion dollars on evidence-based technology assessments (studies on comparative effectiveness). This will certainly bring greater efficiency to the plan, because waste will be prevented in some sectors, and procedures with better defined effectiveness and safety will be included. It will also facilitate the expansion of healthcare coverage to a population of more than 50 million people in the richest country in the world who had been excluded until now.

More information at the website www.htai2011.org.

